# Deep-Sea Target Localization with Entropy Reduction: Sound Ray Bending Correction Based on TOA Time Series Analysis and Joint TOA-AOA Fusion

**DOI:** 10.3390/e28040373

**Published:** 2026-03-25

**Authors:** Yuzhu Kang, Xiaohong Shen, Haiyan Wang, Yongsheng Yan, Tianyi Jia

**Affiliations:** 1 School of Marine Science and Technology, Northwestern Polytechnical University, Xi’an 710072, China; yzkang@mail.nwpu.edu.cn (Y.K.); hywang@nwpu.edu.cn (H.W.); ysyan@nwpu.edu.cn (Y.Y.); 2 Key Laboratory of Ocean Acoustics and Sensing, Ministry of Industry and Information Technology, Xi’an 710072, China; 3 National Key Laboratory of Radar Signal Processing, Xidian University, Xi’an 710071, China; jiatianyi@xidian.edu.cn

**Keywords:** deep-sea target localization, sensor position error, sound ray bending, joint TOA-AOA, closed-form solution, CRLB

## Abstract

Unlike terrestrial environments, the inhomogeneity distribution of underwater sound speed poses significant challenges for underwater ranging and target localization. In the presence of sound ray bending and sensor node position errors in underwater acoustic sensor networks (UASNs), this paper proposes a joint TOA-AOA deep-sea target localization framework based on sound ray bending correction. From the perspective of information theory and time series analysis, the TOA measurements are time series signals carrying target position information, and the entropy-based analysis quantifies the fundamental limit on localization uncertainty. First, based on the TOA time series measurements and combined with the acoustic propagation characteristics of the deep sea, a sound ray bending correction method is adopted to improve the accuracy of slant range measurement. To enhance target localization accuracy, this paper proposes a two-step WLS closed-form solution based on TOA-AOA. To further reduce localization bias, a maximum likelihood estimation (MLE) method based on the Gauss-Newton is also derived. Subsequently, the paper derives and analyzes the Cramér-Rao lower bound (CRLB) for target localization, proving theoretically that jointly using TOA-AOA can improve localization accuracy. Simulations verify the performance of the proposed methods. The slant range estimation method based on sound ray bending correction effectively improves range measurement accuracy. The proposed closed-form solution enhances target localization accuracy, achieving the CRLB accuracy. The Gauss-Newton MLE solution can attain the CRLB accuracy under certain localization geometries and further reduces localization bias.

## 1. Introduction

As an underwater monitoring network system, underwater acoustic sensor networks (UASNs) are increasingly being widely applied in ocean exploration, marine environment monitoring, seabed resource exploration, and military operations due to their advantages, such as simple infrastructure, low equipment costs, and easy deployment [1,2]. Whether for civilian development or military purposes, determining the location of the mission area or target position is the key and prerequisite for the successful execution of the mission. However, due to the severe attenuation of electromagnetic wave signals underwater, the global positioning system (GPS) cannot be applied in such underwater environments [3]. Instead, UASNs can provide a viable solution for underwater target localization [4]. In addition, the positioning errors accumulated by the dead reckoning system or the inertial navigation system during unmanned underwater vehicle (UUV) navigation can be corrected through UASN-assisted localization, thus improving localization accuracy [5,6]. UASNs localization includes self-localization of sensor nodes and target localization. Target localization algorithms need to use the sensor positions and the measurement data from sensors to locate the underwater target, so the position accuracy of sensors will have a significant impact on the performance of underwater target localization algorithms.

Common measurement techniques for UASNs localization include time of arrival (TOA), time difference of arrival (TDOA), received signal strength (RSS), angle of arrival (AOA), Doppler shift, frequency difference of arrival (FDOA), and their combinations [7,8,9,10]. Each of these methods has its advantages and limitations. TOA-based methods require precise time synchronization in the localization system and are suitable for cooperative source localization or active target localization. In comparison, TDOA-based methods are more general in that not only do they not require time synchronization between sensors and target (though time synchronization between sensors is still required), but TOA measurements can also be converted to TDOA measurements. The signal strength or signal energy decays with increasing propagation range. RSS-based methods exploit the characteristic for target localization when the signal transmission power is known and do not require strict time synchronization. However, RSS-based methods are susceptible to multipath interference, resulting in relatively poor localization accuracy. AOA measurement can be obtained by measuring the phase difference of the signal through a sensor array. AOA-based methods only require two sensor nodes to achieve three-dimensional (3D) localization. However, AOA-based methods require each node to have an array of direction-finding capability to obtain both azimuth and elevation angles, and the localization accuracy is highly dependent on the range between the target and the sensor network. When the target is located within or not far from the sensor network, the localization results are accurate. Doppler shift and FDOA measurements require relative motion between the target and the sensor network for estimating moving targets and are usually combined with other types of measurements to improve localization accuracy. Ref. [11] proposed a passive target localization method that combines TDOA and AOA measurements. Compared to using either TDOA or AOA measurements alone, the combined TDOA-AOA localization method can provide better performance with fewer sensors [11,12,13]. In this study, we focus on cooperative target localization and investigate a 3D target localization method based on TOA-AOA measurements to improve localization accuracy.

Generally speaking, the direct relationship between the target position and the measurements is nonlinear and non-convex, making the target localization problem complex. The maximum likelihood estimation (MLE) is an asymptotically efficient estimation method [14]. However, due to the nonlinear and non-convex relationship between the target position and the measurements, it is difficult to obtain a closed-form solution for MLE, or the closed-form solution may not exist at all. If the closed-form solution for MLE cannot be obtained, the grid search method or the numerical iterative method for maximizing the likelihood function can be used to solve the problem [15,16]. However, the grid search method is time-consuming, and numerical iterative algorithms such as gradient descent and Newton’s method require initial values close to the true value to start the iteration, making it difficult to ensure computation time and convergence.

To address this issue, namely to enhance the robustness of the methods and reduce their complexity, refs. [11,17,18] constructed an overdetermined pseudo-linear equation system Au≈h based on the nonlinear relationship between the target position and the measurements, where A and h are constructed from the measurements. Ref. [18] proposed a least squares (LS) localization algorithm for solving the pseudo-linear equation system, achieving relatively good localization accuracy. Considering that both the matrix A and the vector h in the pseudo-linear equation are affected by noise, refs. [11,17] further extends this method to a weighted least-squares (WLS) localization method, which reduces the estimation bias to some extent and has high computational efficiency. However, considering only the first-order Taylor expansion of the noise term is insufficient, as the localization bias of this method increases with the measurement noise power. Therefore, ref. [19] proposed a structured total least squares (STLS) method, which utilizes the special structure of matrix A to further reduce the localization bias caused by noise terms. Although the WLS methods proposed in [17,19] achieve good localization performance and reduce localization bias, and the STLS method in [19] further reduce localization bias, the localization performance of these WLS and STLS methods based on joint TDOA and AOA measurements do not reach the Cramér-Rao lower bound (CRLB) accuracy. Moreover, they all assumed that the sensor positions are accurate, without considering that sensor positions can also be affected by noise in real scenarios.

Due to the inhomogeneous nature of the ocean medium and the uneven distribution of sound speed, sound rays bend as they propagate through the ocean [20,21,22]. In UASNs that rely on TOA or TDOA measurements for localization, using the average sound speed multiplied by TOA or TDOA to estimate the straight-line propagation range or range difference can meet certain localization accuracy requirements [23,24]. However, if the selected average sound speed is inaccurate, it will introduce even greater range errors. Such inaccurate range estimation will further increase the entropy of the subsequent target position estimation process, resulting in a significant degradation of localization accuracy.

To enhance the localization accuracy of cooperative target, considering the impact of sensor position errors and measurement noise on target localization performance, this paper proposes a two-step WLS closed-form solution based on TOA-AOA. First, in order to obtain more accurate range measurements from TOA measurements, this paper adopts a sound ray bending correction method according to the characteristics of deep-sea acoustic propagation to improve ranging precision. This closed-form solution introduces a redundant parameter when constructing the pseudo-linear equations, enabling the derivation of a WLS solution that includes the target position. Subsequently, the relationship between the target position and the redundant parameter is utilized to improve the localization accuracy of the target. To further reduce the target localization bias, this paper also derives an MLE method based on the Gauss-Newton iteration.

The remainder of the paper is organized as follows. Section II proposes a slant range estimation method based on sound ray bending correction. Section III presents the localization scenario and formulates the measurement models. Section IV proposes a deep-sea target localization framework based on sound ray bending correction, and then develops a closed-form solution for target localization. Section V derives and analyzes the CRLB for the target localization. Section VI validates the theory through simulation, and Section VII concludes the paper.

Notations: bold lowercase letters represent column vectors, and bold uppercase letters represent matrices. If (∗) contains noise, ${\left(*\right)}^{o}$ is used to denote the true value of (∗). $I_{\left(*)\times (*\right)}$ represents an identity matrix of size (∗)×(∗), $O_{\left(*)\times (*\right)}$ represents a zero matrix of size (∗)×(∗), and $0_{\left(*\right)}$ represents a zero vector of size (∗)×1. For matrix R, R≽O indicates R is positive semidefinite (PSD).

## 2. Slant Range Estimation

For underwater target localization based on TOA measurements, calculating the straight-line ranges between the target and the nodes by multiplying the average sound speed by the TOA measurements can satisfy certain localization accuracy requirements. However, if the average sound speed is inaccurate, the ranging errors can become significant, subsequently leading to even greater localization error. To ensure high localization accuracy for the target, it is essential to improve the ranging accuracy. This paper proposes a slant range estimation method based on sound ray bending correction. According to the distribution of the underwater sound speed and the characteristics of sound ray propagation, this method employs ray acoustics theory to estimate the precise straight-line range between the node and the target.

### 2.1. Ray Acoustics Theory

In underwater acoustics, ray acoustics is commonly employed as an analytical approach. The inhomogeneous distribution of sound speed in water causes the direction of sound ray propagation to bend. The sound speed in seawater is influenced by temperature, salinity, and pressure, all of which vary with depth and exhibit vertical stratification. Due to the influence of these three factors, the sound speed in seawater also varies with depth and displays vertical stratification characteristics. In engineering practice, after measuring the depth-dependent sound speed profile c(z), c(z) is usually divided into several layers along the depth direction, with the relative sound speed gradient a in each layer being constant. This is known as the stratified medium model [25].

The fundamental principle followed in ray acoustics is Snell’s law [26], which is expressed as

(1) $$\frac{\cos\alpha_{i}}{c_{i}}=\frac{\cos\alpha_{0}}{c_{0}}=\mathrm{constant}$$

where $\alpha_{i}$ represents the grazing angle between the sound ray and the horizontal axis Ox at depth $z_{i}$ (the boundary of the i-th layer), and $c_{i}$ denotes the sound speed at depth $z_{i}$. $\alpha_{0}$ and $c_{0}$ correspond to the grazing angle and sound speed at a specific depth, such as the depth of the sound source where the ray is emitted. Given $\alpha_{0}$ and the vertically stratified distribution of sound speed c(z), the grazing angle of the sound ray at any depth in the ocean can be determined using Snell’s law, thereby defining the propagation direction of sound wave throughout the water column.

**Figure 1:**
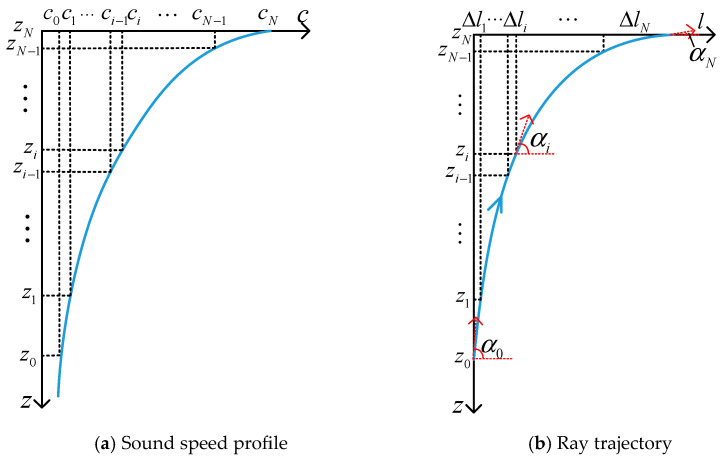
Schematic diagram of sound speed profile and ray trajectory.

In the acoustic localization of a deep-sea target, the target is located below the sensor nodes. The target transmits acoustic signals, and the sound rays emitted from the target bend upward as they propagate to reach the sensor nodes. Figure 1 illustrates the sound speed profile and ray trajectory for a stratified medium model, with the left panel showing the sound speed profile and the right panel depicting the sound ray trajectory.

In Figure 1, l represents the horizontal range of the sound ray propagation. When the sound speed distribution is approximated as a multi-layer constant gradient distribution, the horizontal range and propagation time in each layer can be calculated based on the initial grazing angle $\alpha_{0}$ of the sound ray and the principles of ray acoustics. Let $z_{i}$ and $\alpha_{i}$ denote the depth and grazing angle of the sound ray at the boundary of the i-th layer, respectively. $g_{i}=dc_{i}/dz_{i}$, $\Delta l_{i}$, and $\Delta t_{i}$ represent the sound speed gradient, horizontal range, and propagation time in the i-th layer, respectively. Under the vertically layered model assumption, the total horizontal range and propagation time for an N-layer sound ray are given by:

(2) $$l=\sum_{i=1}^{N}\Delta l_{i}$$

(3) $$t=\sum_{i=1}^{N}\Delta t_{i}$$

where

(4) $$\Delta l_{i}=\frac{\left|z_{i}-z_{i-1}\right|}{\tan\left[\frac{1}{2}\left[\alpha_{i}+\alpha_{i-1}\right]\right]}$$

(5) $$\Delta t_{i}=\left|\frac{1}{g}\ln\left[\frac{\tan(\frac{\alpha_{i}}{2}+\frac{\pi }{4})}{\tan(\frac{\alpha_{i-1}}{2}+\frac{\pi }{4})}\right]\right|$$

From (2) to (5), it can be seen that both the horizontal range traveled by the sound ray and the propagation time are functions of the initial grazing angle $\alpha_{0}$. Given the target depth, node depth, and sound speed profile, there must be a corresponding horizontal range and propagation time.

### 2.2. Slant Range Estimation Method Based on Sound Ray Bending Correction

In deep-sea environments, due to the simple types, small quantity, and easy distinguishability of sound rays, it is feasible to calculate the range between node and target using the ray acoustics theory. Through the interaction between nodes and the target in UASNs, the propagation time of the sound ray can be measured by the nodes. The detailed steps for the slant range estimation method are as follows:

(1) Determine the node depth and target depth, and set the initial grazing angle $\alpha_{0}$.

(2) Combined with the specific sound speed gradient, approximately divide the sound speed distribution into N layers with equal sound speed gradient distribution. Apply Snell’s law and formulas (2) and (3) to calculate the horizontal propagation range l and propagation time t under the condition of $\alpha_{0}$.

(3) Compare the calculated sound ray propagation time t with the measured propagation time $t^{\prime }$. If the difference satisfies the predetermined precision requirement $\delta =\left|t-t^{\prime }\right|\leq Q$, then the calculated l is the required horizontal propagation range of the sound ray. If the difference does not meet the precision requirement, the initial grazing angle needs to be reset. The specific method is: if $t>t^{\prime }$, increase the initial grazing angle, do so; otherwise, decrease it. The bisection method may be utilized for this iterative refinement.

(4) Repeat steps (2) to (3) until the predetermined precision requirement $\delta =\left|t-t^{\prime }\right|\leq Q$ is satisfied. The resulting initial grazing angle is the real initial grazing angle, and the corresponding horizontal range is the real horizontal range traveled by the sound ray.

(5) The slant range is obtained from the horizontal range and depth difference between the node and the target using the Pythagorean theorem.

To facilitate the implementation of the algorithm, the algorithm box for the slant range estimation method based on sound ray bending correction is presented as follows (Algorithm 1).
**Algorithm 1:** Slant range estimation method based on sound ray bending correction**Input**: Sensor depth $z_{s}$, target depth $z_{u}$; Propagation time measurement $t^{\prime }$; Sound speed profile c(z);**Output**: Slant range r;1: Initialize the grazing angle range $\left(\alpha_{\mathrm{start}},\alpha_{\mathrm{end}}\right)$;2: Divide the sound speed profile into N layers;3: **repeat**4:    Under the initial grazing angle $\alpha_{0}=\left(\alpha_{\mathrm{start}}+\alpha_{\mathrm{end}}\right)/2$, calculate the horizontal propagation range l and propagation time t using Formulas (1)–(3);5:   Compare $\delta =\left|t-t^{\prime }\right|$ with the threshold Q;6:   **if** $\delta =\left|t-t^{\prime }\right|\leq Q$ **then**7:    The horizontal propagation range is l;8:   **else**9:    **if** $t>t^{\prime }$ **then**10:     $\alpha_{\mathrm{start}}=\alpha_{0}$, $\alpha_{0}=\left(\alpha_{\mathrm{start}}+\alpha_{\mathrm{end}}\right)/2$;11:    **else**12:     $\alpha_{\mathrm{end}}=\alpha_{0}$, $\alpha_{0}=\left(\alpha_{\mathrm{start}}+\alpha_{\mathrm{end}}\right)/2$;13: **until** $\delta =\left|t-t^{\prime }\right|\leq Q$;14: **return**
$r=\sqrt{l^{2}+{\left(z_{u}-z_{s}\right)}^{2}}$;

## 3. Scenario and Measurement Models

**Figure 2:**
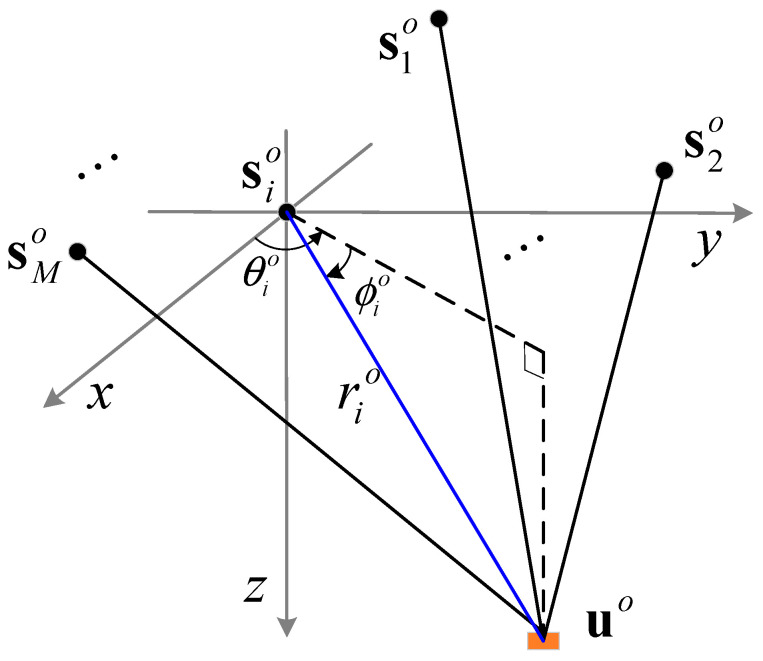
Localization geometry of TOA-AOA measurements in 3D scenario.

In the localization scenario shown in Figure 2, there are M sensors and one target in the 3D Cartesian coordinate system. The true position of the target, denoted as $u^{o}={\left[x^{o},y^{o},z^{o}\right]}^{T}$, is unknown and needs to be estimated using the TOA and AOA measurements of the target signal received by the sensors. The true position of sensor i, denoted as $s_{i}^{o}={\left[x_{i}^{o},y_{i}^{o},z_{i}^{o}\right]}^{T},i=1,2,\cdots ,M$, is also unknown, and the available sensor position $s_{i}={\left[x_{i},y_{i},z_{i}\right]}^{T}$ is inaccurate and can be modeled as

(6) $$s_{i}=s_{i}^{o}+\Delta s_{i}$$

where $\Delta s_{i}$ represents the position error of the sensor i. For notation simplicity, we collect the available sensor positions to form a 3M×1 sensor position vector $s={\left[s_{1}^{T},s_{2}^{T},\cdots ,s_{M}^{T}\right]}^{T}=s^{o}+\Delta s$, where $s^{o}={\left[s_{1}^{oT},s_{2}^{oT},\cdots ,s_{M}^{oT}\right]}^{T}$ is the true sensor position vector and $\Delta s={\left[\Delta s_{1}^{T},\Delta s_{2}^{T},\cdots ,\Delta s_{M}^{T}\right]}^{T}$ is a zero-mean Gaussian random vector with covariance matrix $Q_{s}$.

Due to the difficulty of achieving precise clock synchronization among sensors and between sensors and the target, the measurement process for target localization follows the cooperative localization scheme proposed by Kang [27]. The TOA measurements can be converted into the range measurements between the sensors and the target by applying the sound ray bending correction method. The range measurement from sensor i to the target is given by

(7) $$r_{i}=r_{i}^{o}+n_{ri}$$

where $r_{i}^{o}$ represents the true range, and $n_{ri}$ represents the range measurement noise. We collect the range measurements between M sensors and the target into an M×1 range measurement vector $r={\left[r_{1},r_{2},\cdots ,r_{M}\right]}^{T}=r^{o}+n_{r}$, where $r^{o}={\left[r_{1}^{o},r_{2}^{o},\cdots ,r_{M}^{o}\right]}^{T}$ is the true range vector, and $n_{r}={\left[n_{r1},n_{r2},\cdots ,n_{rM}\right]}^{T}$ is a zero-mean Gaussian random vector with covariance matrix $Q_{r}$.

Each sensor can also measure the angle of the target, obtaining a pair of AOA measurements, denoted as the azimuth and elevation angle pair $\left(\theta_{i},\phi_{i}\right)$. The true AOA pair is $\left(\theta_{i}^{o},\phi_{i}^{o}\right)$, and the AOA values measured by sensor i are modeled as

(8a) $$\theta_{i}=\theta_{i}^{o}+n_{\theta i}$$

(8b) $$\phi_{i}=\phi_{i}^{o}+n_{\phi i}$$

where $n_{\theta i}$ and $n_{\phi i}$ represent the measurement noise of azimuth and elevation angles, respectively. We collect M pairs of AOA measurements into an 2M×1 AOA measurement vector $a={\left[\theta_{1},\phi_{1},\theta_{2},\phi_{2},\cdots ,\theta_{M},\phi_{M}\right]}^{T}=a^{o}+n_{a}$, where $a^{o}={\left[\theta_{1}^{o},\phi_{1}^{o},\theta_{2}^{o},\phi_{2}^{o},\cdots ,\theta_{M}^{o},\phi_{M}^{o}\right]}^{T}$ is the true AOA value vector and $n_{a}={\left[n_{\theta 1},n_{\phi 1},n_{\theta 2},n_{\phi 2},\cdots ,n_{\theta M},n_{\phi M}\right]}^{T}$ is a zero-mean Gaussian random vector with covariance matrix $Q_{a}$.

The noise vectors Δs, $n_{r}$, and $n_{a}$ are assumed to be independent of each other. The range measurement vector **r** and the AOA measurement vector **a** are combined to form an overall measurement vector

(9) $$\kappa ={\left[r^{T},a^{T}\right]}^{T}=\kappa^{o}+n_{\kappa }$$

where $\kappa^{o}={\left[r^{oT},a^{oT}\right]}^{T}$ is the true value corresponding to the measurement vector, and the measurement noise vector $n_{\kappa }={\left[n_{r}^{T},n_{a}^{T}\right]}^{T}$ is a zero-mean Gaussian random vector with covariance matrix $Q_{\kappa }=\mathrm{diag}(Q_{r},Q_{a})$.

According to Figure 2, the relationships between the true values of range and AOA and the true positions of the target and sensors are shown as follows

(10) $$r_{i}^{o}=\left\|u^{o}-s_{i}^{o}\right\|$$

(11) $$\tan(\theta_{i}^{o})=\frac{\sin(\theta_{i}^{o})}{\cos(\theta_{i}^{o})}=\frac{y^{o}-y_{i}^{o}}{x^{o}-x_{i}^{o}}$$

(12) $$\tan(\phi_{i}^{o})=\frac{\sin(\phi_{i}^{o})}{\cos(\phi_{i}^{o})}=\frac{z^{o}-z_{i}^{o}}{\sqrt{{\left(x^{o}-x_{i}^{o}\right)}^{2}+{\left(y^{o}-y_{i}^{o}\right)}^{2}}}$$

where $\theta_{i}^{o}\in (-\pi ,\pi ]$, and $\phi_{i}^{o}\in (-\pi /2,\pi /2)$.

Our objective is to estimate the target position $u^{o}$ as accurately as possible given the noisy sensor position vector s and the measurement vector κ of the range and AOA. From the perspective of time series analysis and information entropy, the core objective of target localization is to extract target position information from high-entropy TOA/AOA measurement time series affected by measurement noise so as to obtain the minimum-entropy estimation of the target position.

## 4. Deep-Sea Target Localization Framework Based on Sound Ray Bending Correction

**Figure 3:**
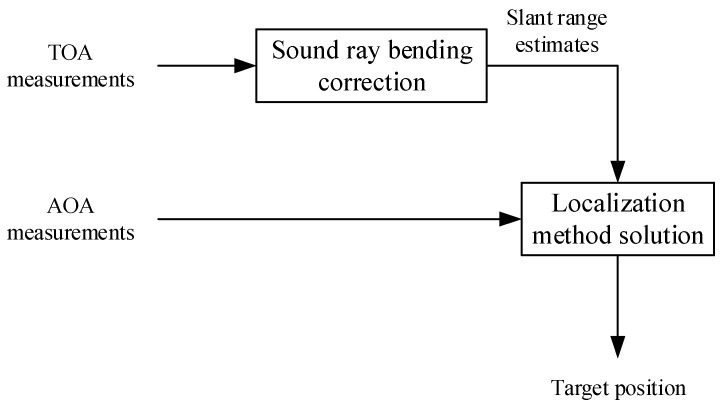
Basic process framework of joint TOA-AOA target localization.

The performance of target localization algorithms is affected by the accuracy of sensor positions and measurements. To improve the ranging accuracy, this paper investigates a slant range estimation method that accounts for ray bending. Based on the measurements of slant range, azimuth angle, and elevation angle, this paper proposes a closed-form solution incorporating range and angle measurements to enhance target localization accuracy. The basic framework from obtaining measurements to solving the target position is illustrated in Figure 3. The proposed localization framework integrates time series analysis and information entropy reduction into the entire process of underwater acoustic measurement and target localization. First, sound ray bending correction is adopted to reduce the entropy of TOA-based range measurement time series. Then, the temporal correlation of TOA/AOA time series is used for measurement preprocessing. Finally, the two-step WLS closed-form solution is applied to achieve high-precision target position estimation with low-entropy data.

In this section, we will first propose an algebraic closed-form solution to localize the target by jointly using range and AOA measurements in the presence of sensor position errors, thereby improving the localization accuracy of the target. The proposed closed-form solution is computationally attractive because, compared to iterative methods, it does not require initial guesses close to the true solution and does not have a local convergence problem. The proposed closed-form solution includes two WLS solutions. However, due to both A and h in the WLS solution being affected by measurement noise, the WLS solution will have a larger bias than the MLE solution. To reduce the bias, the remainder of this section derives a Gauss-Newton MLE solution.

### 4.1. Closed-Form Solution

The proposed closed-form solution consists of two stages. In the first stage, we transform the nonlinear equation system into a pseudo-linear equation system by introducing a redundant parameter and obtain the WLS solution of the unknown parameters. The second stage is to refine the estimation of the target position and improve the target localization accuracy by utilizing the relationship between redundant parameters and target position.

First Stage: This stage exploits the sensor position vector s and the measurement vector κ to estimate the target position $u^{o}$ and the extra variable $u^{oT}u^{o}$ that is assumed to be unrelated to $u^{o}$.

Squaring both sides of (10), substituting $s_{i}^{o}=s_{i}-\Delta s_{i}$ and $r_{i}^{o}=r_{i}-n_{ri}$ into (10), and ignoring the second-order noise terms yield

(13) $$2{\left(u^{o}-s_{i}\right)}^{T}\Delta s_{i}+2r_{i}n_{ri}=r_{i}^{2}-s_{i}^{T}s_{i}+2s_{i}^{T}u^{o}-u^{oT}u^{o}$$

(11) can be transformed into

(14) $$\sin(\theta_{i}^{o})(x^{o}-x_{i}^{o})-\cos(\theta_{i}^{o})(y^{o}-y_{i}^{o})=0$$

It is known that the trigonometric identity $\sin^{2}(\theta_{i}^{o})+\cos^{2}(\theta_{i}^{o})=1$. Multiplying $\sqrt{{\left(x^{o}-x_{i}^{o}\right)}^{2}+{\left(y^{o}-y_{i}^{o}\right)}^{2}}$ by $\sin^{2}(\theta_{i}^{o})+\cos^{2}(\theta_{i}^{o})$ yields

(15) $$\sqrt{{\left(x^{o}-x_{i}^{o}\right)}^{2}+{\left(y^{o}-y_{i}^{o}\right)}^{2}}=\cos(\theta_{i}^{o})(x^{o}-x_{i}^{o})+\sin(\theta_{i}^{o})(y^{o}-y_{i}^{o})$$

Substituting (15) into (12), we obtain

(16) $$\sin(\phi_{i}^{o})\cos(\theta_{i}^{o})(x^{o}-x_{i}^{o})+\sin(\phi_{i}^{o})\sin(\theta_{i}^{o})(y^{o}-y_{i}^{o})-\cos(\phi_{i}^{o})(z^{o}-z_{i}^{o})=0$$

Let $G_{i}^{o}\in {\mathbb{R}}^{3\times 2}$ be

(17) $$G_{i}^{o}=\left[\begin{array}{cc} \sin(\theta_{i}^{o}) & \sin(\phi_{i}^{o})\cos(\theta_{i}^{o})\\ -\cos(\theta_{i}^{o}) & \sin(\phi_{i}^{o})\sin(\theta_{i}^{o})\\ 0 & -\cos(\phi_{i}^{o}) \end{array}\right]$$

According to (14) and (16), we can construct a pseudo-linear equation system about the relationship among the sensor position, the target position, and AOA.

(18) $$G_{i}^{oT}s_{i}^{o}=G_{i}^{oT}u^{o}$$

The column vectors of $G_{i}^{o}$ can be expanded using Taylor series as

(19) $$\begin{array}{cc} G_{i}^{o}(a_{i}^{o}) & \approx G_{i}(a_{i})+\left(\frac{\partial G_{i,1}(a_{i})}{\partial \theta_{i}}(\theta_{i}^{o}-\theta_{i}),\frac{\partial G_{i,2}(a_{i})}{\partial (\theta_{i},\phi_{i})}\left(\begin{array}{l} \theta_{i}^{o}-\theta_{i}\\ \phi_{i}^{o}-\phi_{i} \end{array}\right)\right)\\ & ≜G_{i}(a_{i})+\Delta G_{i}(a_{i}) \end{array}$$

where

$$\frac{\partial G_{i,1}(a_{i})}{\partial \theta_{i}}=\left(\begin{array}{c} \cos(\theta_{i})\\ \sin(\theta_{i})\\ 0 \end{array}\right),\;\frac{\partial G_{i,2}(a_{i})}{\partial (\theta_{i},\phi_{i})}=\left(\begin{array}{cc} -\sin(\phi_{i})\sin(\theta_{i}) & \cos(\phi_{i})\cos(\theta_{i})\\ \sin(\phi_{i})\cos(\theta_{i}) & \cos(\phi_{i})\sin(\theta_{i})\\ 0 & \sin(\phi_{i}) \end{array}\right)$$

Substituting (19) and $s_{i}^{o}=s_{i}-\Delta s_{i}$ into (18), ignoring the second-order noise terms, and simplifying, we have

(20) $$G_{i}^{T}\Delta s_{i}+\Delta G_{i}^{T}(u^{o}-s_{i})=G_{i}^{T}s_{i}-G_{i}^{T}u^{o}$$

Let $c_{1,i}={\left[\cos(\theta_{i}),\sin(\theta_{i}),0\right]}^{T}$, $c_{2,i}={\left[-\sin(\phi_{i})\sin(\theta_{i}),\sin(\phi_{i})\cos(\theta_{i}),0\right]}^{T}$, $c_{3,i}={\left[\cos(\phi_{i})\cos(\theta_{i}),\cos(\phi_{i})\sin(\theta_{i}),\sin(\phi_{i})\right]}^{T}$, then

(21) $$\begin{array}{cc} \Delta G_{i}^{T}(u^{o}-s_{i}) & ={\left(c_{1,i}(\theta_{i}^{o}-\theta_{i}),c_{2,i}(\theta_{i}^{o}-\theta_{i})+c_{3,i}(\phi_{i}^{o}-\phi_{i})\right)}^{T}(u^{o}-s_{i})\\ & =-\left[\begin{array}{cc} c_{1,i}^{T}(u^{o}-s_{i}) & 0\\ c_{2,i}^{T}(u^{o}-s_{i}) & c_{3,i}^{T}(u^{o}-s_{i}) \end{array}\right]\left[\begin{array}{l} n_{\theta i}\\ n_{\phi i} \end{array}\right] \end{array}$$

Combining (13) and (20) in matrix form gives the following

(22) $$e_{1}=h_{1}-A_{1}\eta_{1}^{o}$$

where $h_{1}\in {\mathbb{R}}^{3M}$ and $A_{1}\in {\mathbb{R}}^{3M\times 4}$

(23) $$h_{1}=\left[\begin{array}{c} r_{1}^{2}-s_{1}^{T}s_{1}\\ r_{2}^{2}-s_{2}^{T}s_{2}\\ \vdots \\ r_{M}^{2}-s_{M}^{T}s_{M}\\ G_{1}^{T}s_{1}\\ G_{2}^{T}s_{2}\\ \vdots \\ G_{M}^{T}s_{M} \end{array}\right],\;A_{1}=\left[\begin{array}{cc} -2s_{1}^{T} & 1\\ -2s_{2}^{T} & 1\\ \vdots & \vdots \\ -2s_{M}^{T} & 1\\ G_{1}^{T} & 0_{2}\\ G_{2}^{T} & 0_{2}\\ \vdots & \vdots \\ G_{M}^{T} & 0_{2} \end{array}\right],\;\eta_{1}^{o}=\left[\begin{array}{c} u^{o}\\ u^{oT}u^{o} \end{array}\right]$$

In (22), the error term $e_{1}$ is

(24) $$e_{1}=P_{1}\Delta s+R_{1}n_{\kappa }$$

where $P_{1}\in {\mathbb{R}}^{3M\times 3M}$ and $R_{1}\in {\mathbb{R}}^{3M\times 3M}$ are defined as

(25) $$P_{1}=\left[\begin{array}{cccc} 2{\left(u^{o}-s_{1}\right)}^{T} & 0_{3}^{T} & \cdots & 0_{3}^{T}\\ 0_{3}^{T} & 2{\left(u^{o}-s_{2}\right)}^{T} & \cdots & 0_{3}^{T}\\ \vdots & \vdots & \ddots & \vdots \\ 0_{3}^{T} & 0_{3}^{T} & \cdots & 2{\left(u^{o}-s_{M}\right)}^{T}\\ G_{1}^{T} & O_{2\times 3} & \cdots & O_{2\times 3}\\ O_{2\times 3} & G_{2}^{T} & \cdots & O_{2\times 3}\\ \vdots & \vdots & \ddots & \vdots \\ O_{2\times 3} & O_{2\times 3} & \cdots & G_{M}^{T} \end{array}\right],\;R_{1}=\left[\begin{array}{cccccccc} 2r_{1} & 0 & \cdots & 0 & 0_{2}^{T} & 0_{2}^{T} & \cdots & 0_{2}^{T}\\ 0 & 2r_{2} & \cdots & 0 & 0_{2}^{T} & 0_{2}^{T} & \cdots & 0_{2}^{T}\\ \vdots & \vdots & \ddots & \vdots & \vdots & \vdots & \ddots & \vdots \\ 0 & 0 & \cdots & 2r_{M} & 0_{2}^{T} & 0_{2}^{T} & \cdots & 0_{2}^{T}\\ 0_{2} & 0_{2} & \cdots & 0_{2} & T_{1} & O_{2\times 2} & \cdots & O_{2\times 2}\\ 0_{2} & 0_{2} & \cdots & 0_{2} & O_{2\times 2} & T_{2} & \cdots & O_{2\times 2}\\ \vdots & \vdots & \ddots & \vdots & \vdots & \vdots & \ddots & \vdots \\ 0_{2} & 0_{2} & \cdots & 0_{2} & O_{2\times 2} & O_{2\times 2} & \cdots & T_{M} \end{array}\right]$$

where

$$T_{i}=-\left[\begin{array}{cc} c_{1,i}^{T}(u^{o}-s_{i}) & 0\\ c_{2,i}^{T}(u^{o}-s_{i}) & c_{3,i}^{T}(u^{o}-s_{i}) \end{array}\right]$$

The Equation (22) is nonlinear with respect to $u^{o}$. However, if we assume that $u^{o}$ and $u^{oT}u^{o}$ are independent of each other, then (22) becomes linear with respect to $\eta_{1}^{o}$, and we can apply the WLS method to estimate $\eta_{1}^{o}$

(26) $$\eta_{1}={\left(A_{1}^{T}W_{1}A_{1}\right)}^{-1}A_{1}^{T}W_{1}h_{1}$$

where the weighting matrix $W_{1}=E{\left[e_{1}e_{1}^{T}\right]}^{-1}$. From (24), we can obtain $W_{1}={\left(P_{1}Q_{s}P_{1}^{T}+R_{1}Q_{\kappa }R_{1}^{T}\right)}^{-1}$.

Assuming that the AOA measurement noise and the sensor position error are sufficiently small so that the noise in $A_{1}$ can be ignored, the covariance matrix of $\eta_{1}$ is

(27) $$\mathrm{cov}\left(\eta_{1}\right)={\left(A_{1}^{T}W_{1}A_{1}\right)}^{-1}$$

Second Stage: This stage utilizes the relationship between $u^{o}$ and $u^{oT}u^{o}$ to improve the localization accuracy of the unknown target.

The first three elements of $\eta_{1}$ are the target position, which is the estimator we aim to calculate. In the above WLS, we assume that $u^{o}$ and $u^{oT}u^{o}$ are independent of each other, which inevitably leads to inaccurate solutions. But in fact, they are correlated, so we use this correlation to improve the accuracy of the estimator.

$\Delta \eta_{1}$ represents the estimation error of $\eta_{1}$, and we can have

(28a) $$\eta_{1}(1:3)⊙\eta_{1}(1:3)≃u^{o}⊙u^{o}+2u^{o}⊙\Delta \eta_{1}(1:3)$$

(28b) $$\eta_{1}(4)≃u^{oT}u^{o}+\Delta \eta_{1}(4)$$

where the symbol ⊙ represents the element-by-element multiplication, and $\Delta \eta_{1}(1:3)⊙\Delta \eta_{1}(1:3)$ is ignored. (28a) and (28b) are combined after shifting transformation, and we obtain

(29) $$V\Delta \eta_{1}=h_{2}-A_{2}\eta_{2}^{o}$$

where

(30) $$V=\left[\begin{array}{cccc} 2x^{o} & 0 & 0 & 0\\ 0 & 2y^{o} & 0 & 0\\ 0 & 0 & 2z^{o} & 0\\ 0 & 0 & 0 & 1 \end{array}\right],\;h_{2}=\left[\begin{array}{c} \eta_{1}^{2}\left(1\right)\\ \eta_{1}^{2}\left(2\right)\\ \eta_{1}^{2}\left(3\right)\\ \eta_{1}\left(4\right) \end{array}\right],\;A_{2}=\left[\begin{array}{ccc} 1 & 0 & 0\\ 0 & 1 & 0\\ 0 & 0 & 1\\ 1 & 1 & 1 \end{array}\right],\;\eta_{2}^{o}=u^{o}⊙u^{o}=\left[\begin{array}{c} x^{o2}\\ y^{o2}\\ z^{o2} \end{array}\right]$$

At this point, (29) is linear with respect to $\eta_{2}^{o}$, and the WLS solution of $\eta_{2}^{o}$ is

(31) $$\eta_{2}={\left(A_{2}^{T}W_{2}A_{2}\right)}^{-1}A_{2}^{T}W_{2}h_{2}$$

where the weighting matrix $W_{2}=E{\left[(V\Delta \eta_{1}){\left(V\Delta \eta_{1}\right)}^{T}\right]}^{-1}={\left(V\mathrm{cov}(\eta_{1})V^{T}\right)}^{-1}$, and $\mathrm{cov}\left(\eta_{1}\right)$ is given by (27). The final target position estimate is

(32) $$u=\mathrm{diag}\{\mathrm{sign}[\eta_{1}(1:3)]\}\sqrt{\eta_{2}}$$

In the calculation of the two-step WLS solution, the weighting matrices $W_{1}$ and $W_{2}$ contain the true position $u^{o}$ of the target. We can first set $W_{1}$ as the identity matrix to obtain an initial estimate u, and then recalculate the approximate $W_{1}$ to estimate $\eta_{1}$ using (26) [28]. Subsequently, the u estimate in $\eta_{1}$ can be used to replace the true target position $u^{o}$ in $W_{2}$. The simulation results indicate that the performance degradation caused by such approximation is insignificant.

### 4.2. Gauss-Newton MLE Solution

The data vector $d={\left[\kappa^{T},s^{T}\right]}^{T}$, and the unknown parameter vector $\lambda^{o}={\left[u^{oT},s^{oT}\right]}^{T}$. From (6) and (9), the likelihood function of the data vector d is

(33) $$f(d\left|\lambda^{o}\right.)=\frac{1}{{\left(2\pi \right)}^{6M/2}{\left|Q_{d}\right|}^{1/2}}\exp\{-\frac{1}{2}{\left(d-d^{o}(\lambda^{o})\right)}^{T}Q_{d}^{-1}(d-d^{o}(\lambda^{o}))\}$$

where d is a 6M×1 vector with error covariance matrix $Q_{d}=\mathrm{diag}(Q_{\kappa },Q_{s})$, and its corresponding true value vector $d^{o}(\lambda^{o})$ is a function of $\lambda^{o}$.

The MLE method requires maximizing the likelihood function to solve for $\lambda^{o}$. Directly maximizing (33) is not easy, so the usual approach is to minimize the objective function [29]

(34) $$J_{ML}(\lambda^{o})={\left(d-d^{o}(\lambda^{o})\right)}^{T}Q_{d}^{-1}(d-d^{o}(\lambda^{o}))$$

Assuming $\lambda^{\left(1\right)}$ is the initial value of $\lambda^{o}$, we perform Taylor series expansion of the nonlinear function $d^{o}(\lambda^{o})$ at $\lambda^{\left(1\right)}$, ignoring second-order and higher-order terms, we obtain

(35) $$d^{o}(\lambda^{o})\approx d^{o}(\lambda^{\left(1\right)})+B_{1}(\lambda^{o}-\lambda^{\left(1\right)})$$

where $B_{1}=\frac{\partial d^{o}(\lambda^{o})}{\partial \lambda^{oT}}{|}_{\lambda^{o}=\lambda^{\left(1\right)}}$ is detailed in the Appendix A. Substituting (35) into (34) and solving the equation $\frac{\partial J(\lambda^{o})}{\partial \lambda^{o}}=0_{3(M+1)}$, we obtain

(36) $$\lambda^{\left(2\right)}=\lambda^{\left(1\right)}+{\left(B_{1}^{T}Q_{d}^{-1}B_{1}\right)}^{-1}B_{1}^{T}Q_{d}^{-1}(d-d^{o}(\lambda^{\left(1\right)}))$$

By repeating the above steps with $\lambda^{\left(2\right)}$ as the initial value of $\lambda^{o}$, the Gauss-Newton iterative equation can be obtained.

(37) $$\lambda^{\left(k+1\right)}=\lambda^{\left(k\right)}+{\left(B_{1}^{T}Q_{d}^{-1}B_{1}\right)}^{-1}B_{1}^{T}Q_{d}^{-1}(d-d^{o}(\lambda^{\left(k\right)}))$$

where k=1,2,⋯ represents the number of iterations. The target position estimated by the two-step WLS method [30] and the noisy sensor positions can be used as the initial value $\lambda^{\left(1\right)}$. The iteration stops when $\left\|\lambda^{\left(k+1\right)}-\lambda^{\left(k\right)}\right\|$ is less than a certain threshold.

## 5. CRLB and Analysis

The CRLB establishes a lower bound on the error covariance matrix of unbiased estimators, which is often used as a benchmark for evaluating the performance of unbiased estimators. Below, we derive the CRLB of the target position $u^{o}$ and theoretically analyze whether joint TOA-AOA can improve the localization accuracy of the target.

### 5.1. CRLB

The data vector is $d={\left[\kappa^{T},s^{T}\right]}^{T}$, and the unknown parameter vector is $\lambda^{o}={\left[u^{oT},s^{oT}\right]}^{T}$. Because $n_{\kappa }$ and Δs are Gaussian distributed and independent of each other, the logarithm of the probability density function of d (with $\lambda^{o}$ as the parameter) is

(38) $$\ln p(d;\lambda^{o})\;=C-\frac{1}{2}{\left(\kappa -\kappa^{o}\right)}^{T}Q_{\kappa }^{-1}(\kappa -\kappa^{o})-\frac{1}{2}{\left(s-s^{o}\right)}^{T}Q_{s}^{-1}(s-s^{o})$$

where C is a constant.

The CRLB of $\lambda^{o}$ [31] is

(39) $$\mathrm{CRLB}(\lambda^{o}{)=\mathrm{FIM}}^{-1}(\lambda^{o})$$

where $\mathrm{FIM}(\lambda^{o})$ is the Fisher information matrix of the unknown parameter vector $\lambda^{o}$. The $\mathrm{FIM}(\lambda^{o})$ is

(40) $$\begin{array}{l} \mathrm{FIM}(\lambda^{o})=-E\left[\frac{\partial^{2}\ln p(d;\lambda^{o})}{\partial \lambda^{o}\partial \lambda^{oT}}\right]=-E\left[\begin{array}{cc} \frac{\partial^{2}\ln p(d;\lambda^{o})}{\partial u^{o}\partial u^{oT}} & \frac{\partial^{2}\ln p(d;\lambda^{o})}{\partial u^{o}\partial s^{oT}}\\ \frac{\partial^{2}\ln p(d;\lambda^{o})}{\partial s^{o}\partial u^{oT}} & \frac{\partial^{2}\ln p(d;\lambda^{o})}{\partial s^{o}\partial s^{oT}} \end{array}\right]\\ \;\;\;\;\;\;\;\;\;\;\;\;\;\;\;=\left[\begin{array}{cc} X & Y\\ Y^{T} & Z \end{array}\right] \end{array}$$

where

(41) $$X=\frac{\partial \kappa^{oT}}{\partial u^{o}}Q_{\kappa }^{-1}{\left(\frac{\partial \kappa^{oT}}{\partial u^{o}}\right)}^{T},\;Y=\frac{\partial \kappa^{oT}}{\partial u^{o}}Q_{\kappa }^{-1}{\left(\frac{\partial \kappa^{oT}}{\partial s^{o}}\right)}^{T}\;Z=\frac{\partial \kappa^{oT}}{\partial s^{o}}Q_{\kappa }^{-1}{\left(\frac{\partial \kappa^{oT}}{\partial s^{o}}\right)}^{T}+Q_{s}^{-1}$$

where $\frac{\partial \kappa^{oT}}{\partial u^{o}}$ and $\frac{\partial \kappa^{oT}}{\partial s^{o}}$ are respectively

(42a) $$\frac{\partial \kappa^{oT}}{\partial u^{o}}=\left[c_{1},c_{2},\cdots ,c_{M},D_{1},D_{2},\cdots ,D_{M}\right]$$

(42b) $$\frac{\partial \kappa^{oT}}{\partial s^{o}}=[e_{1},e_{2},\cdots ,e_{M},F_{1},F_{2},\cdots ,F_{M}]$$

where $c_{i}\in {\mathbb{R}}^{3\times 1}$, $D_{i}\in {\mathbb{R}}^{3\times 2}$, $e_{i}\in {\mathbb{R}}^{3M\times 1}$, and $F_{i}\in {\mathbb{R}}^{3M\times 2}$, i=1,2,⋯,M are respectively

(43) $$c_{i}=\frac{u^{o}-s_{i}^{o}}{r_{i}^{o}}$$

(44) $$D_{i}=\left[\begin{array}{cc} \frac{y_{i}^{o}-y^{o}}{l_{i}^{o2}} & -\frac{\left(x^{o}-x_{i}^{o})(z^{o}-z_{i}^{o}\right)}{r_{i}^{o2}l_{i}^{o}}\\ \frac{x^{o}-x_{i}^{o}}{l_{i}^{o2}} & -\frac{\left(y^{o}-y_{i}^{o})(z^{o}-z_{i}^{o}\right)}{r_{i}^{o2}l_{i}^{o}}\\ 0 & \frac{l_{i}^{o}}{r_{i}^{o2}} \end{array}\right]$$

(45) $$e_{i}={\left[0_{3(i-1)}^{T},-c_{i}^{T},0_{3(M-i)}^{T}\right]}^{T}$$

(46) $$F_{i}={\left[O_{3(i-1)\times 2}^{T},-D_{i}^{T},O_{3(M-i)\times 2}^{T}\right]}^{T}$$

The CRLB of the target position $u^{o}$ corresponds to the upper left 3×3 submatrix of the inverse matrix of the $\mathrm{FIM}(\lambda^{o})$. Applying the partitioned matrix inversion formula [32], we obtain

(47) $$\mathrm{CRLB}(u^{o})=X^{-1}+X^{-1}Y{\left(Z-Y^{T}X^{-1}Y\right)}^{-1}Y^{T}X^{-1}$$

where $X^{-1}$ represents the CRLB of the target position when there is no error in the sensor positions, and the second term on the right is the increase in the CRLB due to the sensor position errors.

### 5.2. Analysis

**Proposition**: The joint use of TOA and AOA measurements can improve the localization accuracy of the target compared to using TOA or AOA measurements alone.

**Proof**: For (38), unlike (40), when deriving the FIM by treating $\lambda^{o}$ as a whole, we obtain

(48) $${\mathrm{FIM}}_{\mathrm{TOA}/\mathrm{AOA}}(\lambda^{o})={\left(\frac{\partial \kappa^{o}}{\partial \lambda^{oT}}\right)}^{T}Q_{\kappa }^{-1}\frac{\partial \kappa^{o}}{\partial \lambda^{oT}}+{\left(\frac{\partial s^{o}}{\partial \lambda^{oT}}\right)}^{T}Q_{s}^{-1}\frac{\partial s^{o}}{\partial \lambda^{oT}}$$

When only TOA or AOA measurements are available, the FIMs are respectively

(49a) $${\mathrm{FIM}}_{\mathrm{TOA}}(\lambda^{o})={\left(\frac{\partial r^{o}}{\partial \lambda^{oT}}\right)}^{T}Q_{r}^{-1}\frac{\partial r^{o}}{\partial \lambda^{oT}}+{\left(\frac{\partial s^{o}}{\partial \lambda^{oT}}\right)}^{T}Q_{s}^{-1}\frac{\partial s^{o}}{\partial \lambda^{oT}}$$

(49b) $${\mathrm{FIM}}_{\mathrm{AOA}}(\lambda^{o})={\left(\frac{\partial a^{o}}{\partial \lambda^{oT}}\right)}^{T}Q_{a}^{-1}\frac{\partial a^{o}}{\partial \lambda^{oT}}+{\left(\frac{\partial s^{o}}{\partial \lambda^{oT}}\right)}^{T}Q_{s}^{-1}\frac{\partial s^{o}}{\partial \lambda^{oT}}$$

Observing (48), (49a), and (49b), it can be seen that the three expressions share a common term ${\left(\frac{\partial s^{o}}{\partial \lambda^{oT}}\right)}^{T}Q_{s}^{-1}\frac{\partial s^{o}}{\partial \lambda^{oT}}$. Let $F_{\kappa }={\left(\frac{\partial \kappa^{o}}{\partial \lambda^{oT}}\right)}^{T}Q_{\kappa }^{-1}\frac{\partial \kappa^{o}}{\partial \lambda^{oT}}$, $F_{r}={\left(\frac{\partial r^{o}}{\partial \lambda^{oT}}\right)}^{T}Q_{r}^{-1}\frac{\partial r^{o}}{\partial \lambda^{oT}}$, and $F_{a}={\left(\frac{\partial a^{o}}{\partial \lambda^{oT}}\right)}^{T}Q_{a}^{-1}\frac{\partial a^{o}}{\partial \lambda^{oT}}$.

Appendix B proves that $F_{\kappa }{=F}_{r}+F_{a}$. Subtracting (49a) and (49b) from (48), respectively, yields

(50a) $${\mathrm{FIM}}_{\mathrm{TOA}/\mathrm{AOA}}(\lambda^{o})-{\mathrm{FIM}}_{\mathrm{TOA}}(\lambda^{o})=F_{a}$$

(50b) $${\mathrm{FIM}}_{\mathrm{TOA}/\mathrm{AOA}}(\lambda^{o})-{\mathrm{FIM}}_{\mathrm{AOA}}(\lambda^{o})=F_{r}$$

Appendix C proves that $F_{r}$ and $F_{a}$ are positive semidefinite matrices. Therefore,

(51a) $${\mathrm{FIM}}_{\mathrm{TOA}}^{-1}(\lambda^{o})≽{\mathrm{FIM}}_{\mathrm{TOA}/\mathrm{AOA}}^{-1}(\lambda^{o})$$

(51b) $${\mathrm{FIM}}_{\mathrm{AOA}}^{-1}(\lambda^{o})≽{\mathrm{FIM}}_{\mathrm{TOA}/\mathrm{AOA}}^{-1}(\lambda^{o})$$

The CRLB of the target position $u^{o}$ is composed of elements from the inverse of the FIM:

(52a) $${\mathrm{CRLB}}_{\mathrm{TOA}}(u^{o})={\left[{\mathrm{FIM}}_{\mathrm{TOA}}^{-1}(\lambda^{o})\right]}_{1,1}+{\left[{\mathrm{FIM}}_{\mathrm{TOA}}^{-1}(\lambda^{o})\right]}_{2,2}+{\left[{\mathrm{FIM}}_{\mathrm{TOA}}^{-1}(\lambda^{o})\right]}_{3,3}$$

(52b) $${\mathrm{CRLB}}_{\mathrm{AOA}}(u^{o})={\left[{\mathrm{FIM}}_{\mathrm{AOA}}^{-1}(\lambda^{o})\right]}_{1,1}+{\left[{\mathrm{FIM}}_{\mathrm{AOA}}^{-1}(\lambda^{o})\right]}_{2,2}+{\left[{\mathrm{FIM}}_{\mathrm{AOA}}^{-1}(\lambda^{o})\right]}_{3,3}$$

(52c) $${\mathrm{CRLB}}_{\mathrm{TOA}/\mathrm{AOA}}(u^{o})={\left[{\mathrm{FIM}}_{\mathrm{TOA}/\mathrm{AOA}}^{-1}(\lambda^{o})\right]}_{1,1}\;+{\left[{\mathrm{FIM}}_{\mathrm{TOA}/\mathrm{AOA}}^{-1}(\lambda^{o})\right]}_{2,2}+{\left[{\mathrm{FIM}}_{\mathrm{TOA}/\mathrm{AOA}}^{-1}(\lambda^{o})\right]}_{3,3}$$

Applying (51) to (52), we obtain

(53a) $${\mathrm{CRLB}}_{\mathrm{TOA}}(u^{o})\geq {\mathrm{CRLB}}_{\mathrm{TOA}/\mathrm{AOA}}(u^{o})$$

(53b) $${\mathrm{CRLB}}_{\mathrm{AOA}}(u^{o})\geq {\mathrm{CRLB}}_{\mathrm{TOA}/\mathrm{AOA}}(u^{o})$$

Compared to using only one type of measurement for target localization, the Fisher information matrix for joint TOA and AOA measurement localization is “larger”, resulting in a lower CRLB of the target position and higher localization accuracy. Due to the availability of more measurements, the localization accuracy is less affected by measurement noise, leading to stronger robustness.

## 6. Simulation

To validate the performance of the target localization methods proposed in Section IV, this section compares the performance of the proposed methods with those of the target localization methods in [19,30], and also uses the CRLB of the target localization as a benchmark for performance evaluation.

In the simulated localization scenario, there are M=6 sensors and one target to be located. The horizontal positions of the sensors are randomly distributed within a circle with a radius of 500 m centered at (0,0) m, and the depth coordinates of the sensors follow a uniform distribution within [0,100] m. The horizontal position of the target is randomly distributed within a circle with a radius of 1000 m centered at (0,0) m, and the depth coordinate of the target follows a uniform distribution within [0,1000] m. In the simulation, the noisy sensor positions s are generated by adding zero-mean Gaussian noise with covariance matrix $Q_{s}=\sigma_{s}^{2}I_{3M\times 3M}$ to the true values, where $\sigma_{s}^{2}$ is the sensor position error power. The range measurements r between the target and sensors and AOA measurements a of the target are generated in a similar manner, with covariance matrices $Q_{r}=\sigma_{r}^{2}I_{M\times M}$ and $Q_{a}=\sigma_{A}^{2}I_{2M\times 2M}$, where $\sigma_{r}^{2}$ and $\sigma_{A}^{2}$ are the range measurement noise power and AOA measurement noise power, respectively. In the simulation of the Gauss-Newton MLE solution, the iteration stopping condition is $\left\|\lambda^{\left(k+1\right)}-\lambda^{\left(k\right)}\right\|<10^{-8}$.

We use the root mean square error (RMSE) and the bias to evaluate the performance of these target localization methods. The RMSE and bias of the target position estimate u are defined as

(54) $$\mathrm{RMSE}(u)=\sqrt{\frac{1}{L}\sum_{k=1}^{L}{\left\|u_{k}-u^{o}\right\|}_{2}^{2}}$$

(55) $$\mathrm{bias}(u)={\left\|\frac{1}{L}\sum_{k=1}^{L}u_{k}-u^{o}\right\|}_{2}$$

where $u_{k}$ is the target position estimate at ensemble k, and L=5000 is the number of ensemble runs.

### 6.1. Simulation Experiment of Slant Range Estimation Based on Sound Ray Bending Correction

**Figure 4:**
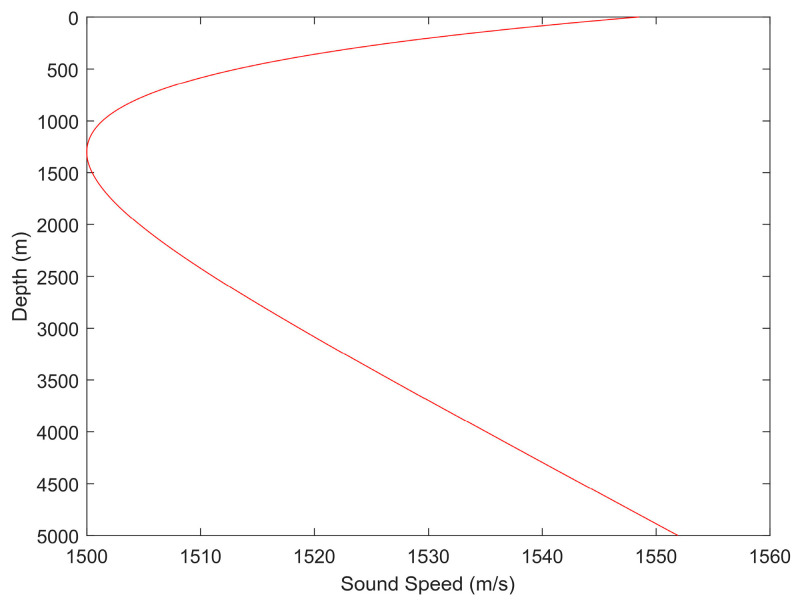
The Munk sound speed profile in deep sea.

This paper proposes a closed-form solution for target localization by jointly using TOA and AOA measurements. To improve the localization accuracy of deep-sea targets, a sound ray bending correction method is employed for slant range estimation based on the acoustic propagation characteristics of the deep sea, thereby enhancing the accuracy of slant range estimation. Since this study focuses on underwater acoustic localization for deep-sea targets, the Munk sound speed profile is adopted, as shown in Figure 4, with a water depth of 5000 m.

**Figure 5:**
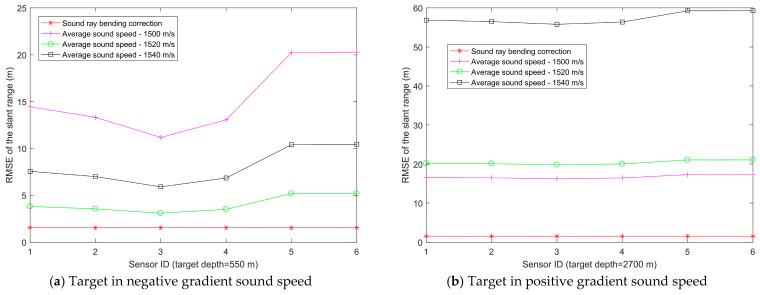
Variation on slant range estimation errors between target and 6 sensors.

**Figure 6:**
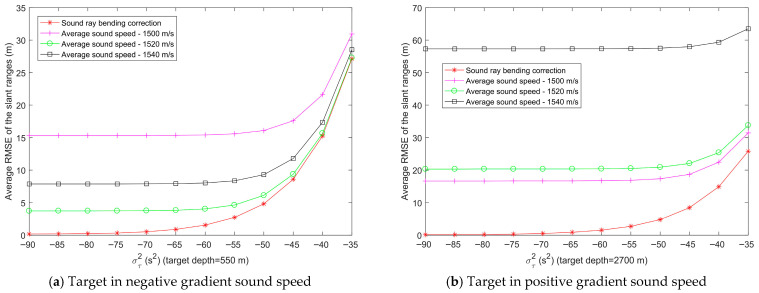
Variation in the average slant range estimation errors between the target and 6 sensors with time delay measurement noise power.

In Figure 4, the Munk sound speed profile can be roughly divided into two segments: the upper segment exhibits a negative gradient sound speed, while the lower segment exhibits a positive gradient sound speed. To verify that the proposed slant range estimation method based on sound ray bending correction is applicable to various sound speed distributions, the target is placed at depths corresponding to the negative gradient and positive gradient sound speed regions, respectively, in the simulations. There are 6 sensor nodes, all deployed at a depth of 5 m, with their horizontal two-dimensional (2D) coordinates of ${\left[300,400\right]}^{T}$ m, ${\left[400,150\right]}^{T}$ m, ${\left[300,500\right]}^{T}$ m, ${\left[350,200\right]}^{T}$ m, ${\left[-100,-100\right]}^{T}$ m, and ${\left[200,-300\right]}^{T}$ m, respectively. The horizontal 2D coordinate of the target is ${\left[600,650\right]}^{T}$ m, and the depths are set to 550 m and 2700 m, respectively. We use Bellhop to simulate the acoustic ray propagation time between the sensors and the target [33,34]. Figure 5 shows the variation in slant range estimation errors for each method across different sensors, and Figure 6 presents the variation in the average slant range estimation errors across the 6 sensors with the time delay measurement noise power for each method. For the average sound speed method used for comparison, the average sound speeds used in the slant range estimation are taken as 1500 m/s, 1520 m/s, and 1540 m/s, respectively. Figure 5a and Figure 6a correspond to a target depth of 550 m, while Figure 5b and Figure 6b correspond to a target depth of 2700 m.

In Figure 5, the time delay measurement noise power $\sigma_{\tau }^{2}$ is 10^−6^ s^2^. All sensor nodes have the same depth difference to the target but different horizontal ranges. The RMSE of the slant range estimation using the average sound speed method differs with the selected value of the average sound speed, and the RMSE also varies across sensors. If the average sound speed is inaccurately selected, the slant range estimation error will be significantly large. In contrast, the slant range estimation based on sound ray bending correction achieves the smallest RMSE, with minimal variation in RMSE across different sensors.

In Figure 6, the average RMSE of slant range estimation for both the average sound speed method and the sound ray bending correction method increases with the growth of the time delay measurement noise power $\sigma_{\tau }^{2}$. Within the tested range of time delay measurement noise levels, the slant range estimation based on sound ray bending correction exhibits the smallest RMSE. Based on the analysis of Figure 5 and Figure 6, it can be observed that the slant range estimation using sound ray bending correction achieves the best performance and remains applicable whether the target is located in the positive or negative gradient sound speed segment.

### 6.2. The Effect of Errors on the Performance of Localization Algorithms

To show a wider range of the tested noise levels, the noise levels are represented on a logarithmic scale. To dynamically display a larger range of the localization results, the RMSE and bias are also represented on a logarithmic scale. The ranges of sensor position error power $\sigma_{s}^{2}$, range measurement noise power $\sigma_{r}^{2}$, and AOA measurement noise power $\sigma_{A}^{2}$ are all set from −60 to 20, corresponding to 10^−6^ m^2^ to 10^4^ m^2^, 10^−6^ m^2^ to 10^4^ m^2^, and 10^−6^ deg^2^ to 10^2^ deg^2^, respectively. This wide range of noise power settings covers the low-entropy to high-entropy states of TOA/AOA measurement time series and sensor positions, which can fully verify the performance of the proposed method under different levels of measurement data uncertainty and sensor position uncertainty, and is more consistent with the real underwater acoustic environment, where the entropy of measurement time series and sensor positions varies significantly.

**Figure 7:**
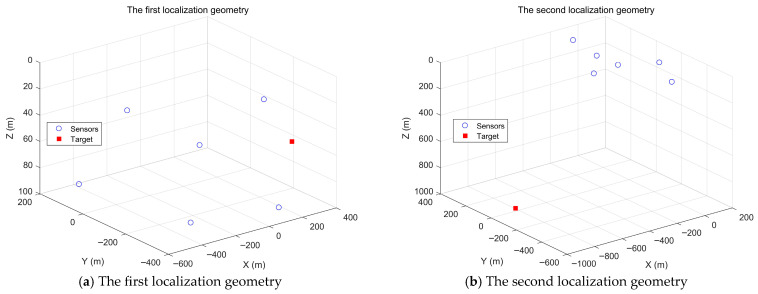
Two randomly generated localization geometries.

**Figure 8:**
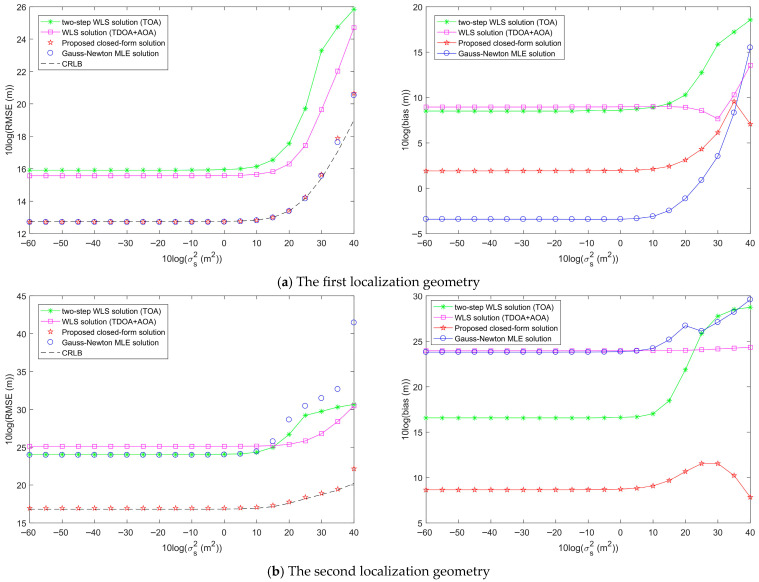
Comparison of the target localization performance of the proposed method and other methods when the sensor position error power $\sigma_{s}^{2}$ increases, $\sigma_{r}^{2}$ is fixed at 10^2^ m^2^, and $\sigma_{A}^{2}$ is fixed at 1 deg^2^.

**Figure 9:**
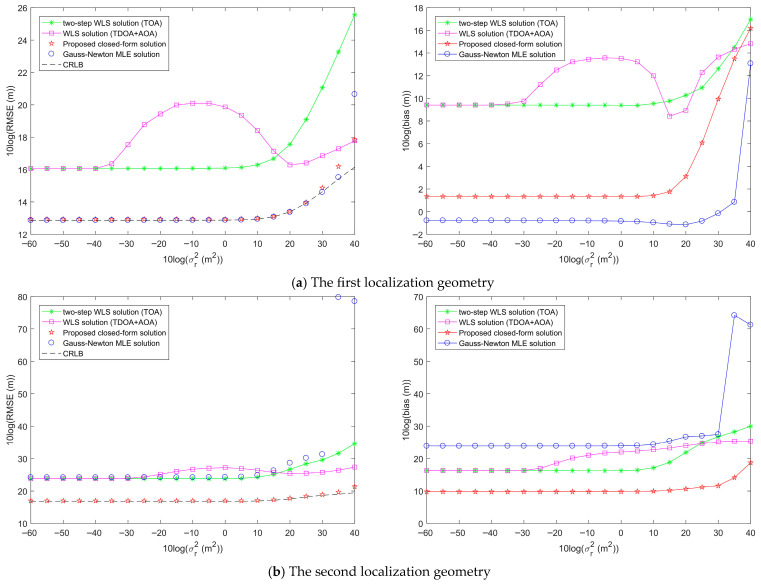
Comparison of the target localization performance of the proposed method and other methods when the range measurement noise power $\sigma_{r}^{2}$ increases, $\sigma_{s}^{2}$ is fixed at 10^2^ m^2^, and $\sigma_{A}^{2}$ is fixed at 1 deg^2^.

**Figure 10:**
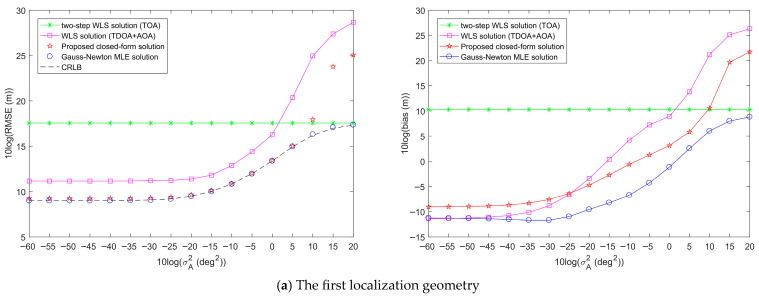

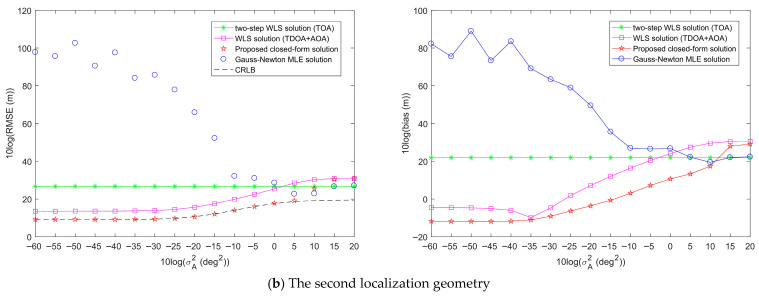
Comparison of the target localization performance of the proposed method and other methods when the AOA measurement noise power $\sigma_{A}^{2}$ increases, $\sigma_{s}^{2}$ is fixed at 10^2^ m^2^, and $\sigma_{r}^{2}$ is fixed at 10^2^ m^2^.

First, two network geometries and target positions are randomly generated to simulate the performance of the proposed target localization methods, as shown in Figure 7. Figure 8 shows the target localization performance of the proposed methods when $\sigma_{r}^{2}$ is fixed at 10^2^ m^2^, $\sigma_{A}^{2}$ is fixed at 1 deg^2^, and $\sigma_{s}^{2}$ gradually increases. Figure 9 demonstrates the target localization performance of the proposed methods when $\sigma_{s}^{2}$ is fixed at 10^2^ m^2^, $\sigma_{A}^{2}$ is fixed at 1 deg^2^, and $\sigma_{r}^{2}$ gradually increases. Figure 10 illustrates the target localization performance of the proposed methods when $\sigma_{s}^{2}$ is fixed at 10^2^ m^2^, $\sigma_{r}^{2}$ is fixed at 10^2^ m^2^, and $\sigma_{A}^{2}$ gradually increases.

Figure 8a presents the target localization results for the first localization geometry. The RMSEs of all methods increase with the increase in sensor position error power $\sigma_{s}^{2}$. The proposed closed-form solution and the Gauss-Newton MLE solution both reach the CRLB accuracy when the sensor position error power $\sigma_{s}^{2}$ is less than 10^3^. As $\sigma_{s}^{2}$ continues to increase, the proposed closed-form solution and the Gauss-Newton MLE solution slowly deviate from the CRLB accuracy, but their RMSEs remain lower than those of the two-step WLS solution and the WLS solution. The two-step WLS solution only uses range measurements to estimate the target position. Although the WLS solution jointly utilizes TDOA and AOA measurements, it does not consider sensor position errors and performs only a single WLS estimation. Therefore, the RMSEs of both methods are greater than those of the proposed methods. From the bias plot in Figure 8a, it can be seen that the biases of the two-step WLS solution and the WLS solution are larger than those of the proposed closed-form solution and the Gauss-Newton MLE solution. The biases of the proposed closed-form solution and the Gauss-Newton MLE solution are not significantly different, with the bias of the Gauss-Newton MLE solution being almost consistently the smallest.

Figure 8b shows the target localization results for the second localization geometry. The RMSEs of all methods increase with the increase in sensor position error power $\sigma_{s}^{2}$. The proposed closed-form solution reaches the CRLB accuracy when the sensor position error power $\sigma_{s}^{2}$ is less than 10^4^. The RMSEs of the Gauss-Newton MLE solution, two-step WLS solution, and WLS solution are all greater than that of the proposed closed-form solution, and none of them achieve the CRLB accuracy. Unlike Figure 8a, the Gauss-Newton MLE solution does not reach the CRLB accuracy in the second localization geometry. From the bias plot in Figure 8b, it is evident that the biases of the Gauss-Newton MLE solution and WLS solution are not significantly different, and both are larger than those of the two-step WLS solution and the proposed closed-form solution, with the bias of the proposed closed-form solution being the lowest.

From the analysis of Figure 8a,b, it is evident that in both localization geometries, the RMSE and bias of the proposed closed-form solution are lower than those of the two-step WLS solution and WLS solution. When the sensor position error power $\sigma_{s}^{2}$ is below a certain large threshold, which is related to the localization geometry, the proposed closed-form solution can reach the CRLB accuracy, and its localization performance is superior to the other two methods. The Gauss-Newton MLE solution reaches the CRLB accuracy only in the first localization geometry, and its corresponding bias is lower than that of the proposed closed-form solution. Therefore, the Gauss-Newton MLE solution can exhibit excellent localization performance only in certain geometries. By analyzing Figure 9 and Figure 10, we can draw conclusions similar to those of Figure 8.

From the analysis of Figure 8, Figure 9 and Figure 10, it is observed that the localization geometry significantly impacts the localization performance of the methods. To mitigate the impact of sensor network geometry and the relative target position on algorithm performance evaluation, a total of 100 random network geometries and target positions are used to obtain the average RMSE and bias.

**Figure 11:**
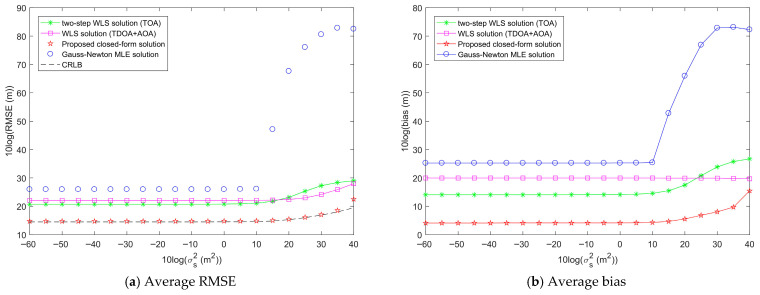
Comparison of the average target localization performance of the proposed method and other methods when the sensor position error power $\sigma_{s}^{2}$ increases, $\sigma_{r}^{2}$ is fixed at 10^2^ m^2^, and $\sigma_{A}^{2}$ is fixed at 1 deg^2^ over 100 random localization geometries.

Figure 11 presents the average RMSE and bias of the proposed method as $\sigma_{s}^{2}$ increases, with $\sigma_{r}^{2}$ fixed at 10^2^ m^2^ and $\sigma_{A}^{2}$ fixed at 1 deg^2^. From Figure 11a, it is evident that the average RMSEs of the Gauss-Newton MLE solution, two-step WLS solution, and WLS solution are all higher than that of the proposed closed-form solution. The proposed closed-form solution reaches the CRLB accuracy when $\sigma_{s}^{2}$ is less than 10^4^ m^2^. From Figure 11b, it is clear that the average bias of the proposed closed-form solution is still lower than those of the other three methods. Based on the above analysis, it can be concluded that the proposed closed-form solution has the best target localization performance within the tested range of sensor position error power.

**Figure 12:**
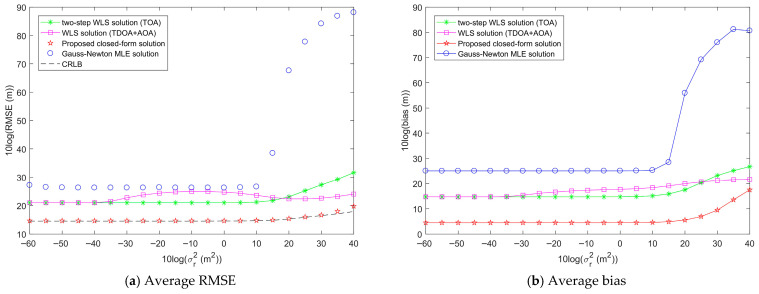
Comparison of the average target localization performance of the proposed method and other methods when the range measurement noise power $\sigma_{r}^{2}$ increases, $\sigma_{s}^{2}$ is fixed at 10^2^ m^2^, and $\sigma_{A}^{2}$ is fixed at 1 deg^2^ over 100 random localization geometries.

Figure 12 shows the average RMSE and bias of the proposed method as $\sigma_{r}^{2}$ increases, with $\sigma_{s}^{2}$ fixed at 10^2^ m^2^ and $\sigma_{A}^{2}$ fixed at 1 deg^2^. From Figure 12a, it can be observed that the average RMSEs of the Gauss-Newton MLE solution, two-step WLS solution, and WLS solution are all higher than that of the proposed closed-form solution. The proposed closed-form solution reaches the CRLB accuracy when $\sigma_{r}^{2}$ is less than 10^3.5^ m^2^. The average RMSE of the Gauss-Newton MLE solution has a very high jump when $\sigma_{r}^{2}$ is equal to 10^1.5^ m^2^, and thus it is no longer viable for target localization. From Figure 12b, it can be seen that the average bias of the proposed closed-form solution is still lower than those of the other three methods. Based on the above analysis, it can be concluded that within the tested range of measurement noise power, the proposed closed-form solution exhibits the best target localization performance.

**Figure 13:**
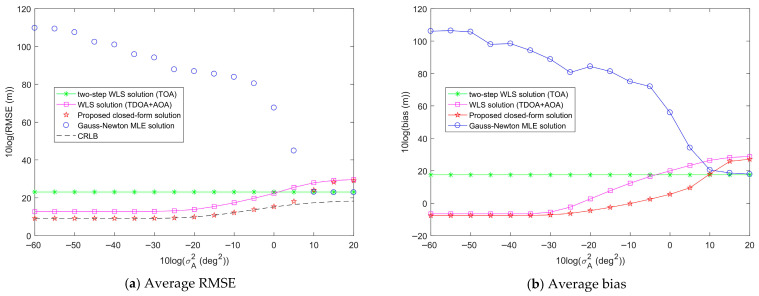
Comparison of the average target localization performance of the proposed method and other methods when the AOA measurement noise power $\sigma_{A}^{2}$ increases, $\sigma_{s}^{2}$ is fixed at 10^2^ m^2^, and $\sigma_{r}^{2}$ is fixed at 10^2^ m^2^ over 100 random localization geometries.

Figure 13 illustrates the average RMSE and bias of the proposed method as $\sigma_{A}^{2}$ increases, with $\sigma_{s}^{2}$ fixed at 10^2^ m^2^ and $\sigma_{r}^{2}$ fixed at 10^2^ m^2^. From Figure 13a, it can be seen that when $\sigma_{A}^{2}$ is less than 10^0.5^ deg^2^, the average RMSE of the proposed closed-form solution is smaller than those of the Gauss-Newton MLE solution, the two-step WLS solution, and WLS solution, achieving the CRLB accuracy. As $\sigma_{A}^{2}$ continues to increase, the average RMSE of the proposed closed-form solution slowly deviates from the CRLB accuracy and gradually tends to be stable, and its average RMSE at this point is higher than that of the two-step WLS solution. Similarly, in Figure 13b, when $\sigma_{A}^{2}$ is less than 10 deg^2^, the average bias of the proposed closed-form solution is smaller than those of the Gauss-Newton MLE solution, two-step WLS solution, and WLS solution. As $\sigma_{A}^{2}$ continues to increase, the average bias of the proposed closed-form solution slowly increases and gradually tends to be stable, and its average bias at this point is higher than those of the Gauss-Newton MLE solution and the two-step WLS solution. It can also be seen that the average RMSE and bias of the two-step WLS solution do not increase with the increase of $\sigma_{A}^{2}$, and remain constant. This is because the two-step WLS solution does not use AOA measurements for target localization, so the AOA measurement noise has no effect on the localization performance.

Through the analysis of Figure 11, Figure 12 and Figure 13, we can see that, except for the two-step WLS solution whose localization performance is not affected by AOA measurement noise, sensor position error, range measurement noise, and AOA measurement noise all have an impact on the performance of the target localization methods. The average RMSE and bias of each method will increase with the increase in noise. In all tested noise ranges, the proposed closed-form solution can achieve the CRLB accuracy and performs the best when the noise is below a certain threshold. For sensor position error and range measurement noise, the average RMSE and bias of the proposed closed-form solution are lower than those of other methods, respectively. For AOA measurement noise, the average RMSE and bias of the proposed closed-form solution are lower than those of other methods when the AOA measurement noise power is below the threshold. In addition, it is obvious that sensor position error, range measurement noise, and AOA measurement noise have different effects on the localization performance of the methods. AOA measurement noise has a greater impact on the localization performance of the methods than sensor position error and range measurement noise.

Inspired by this observation, when jointly using range and AOA measurements for target localization, enhancing the accuracy of these measurements can help improve the target localization performance. When the AOA measurement noise exceeds a certain threshold, it will lead to a significant degradation in the performance of the localization methods, losing the advantage of jointly using range and AOA measurements for localization.

### 6.3. The Robustness of Localization Methods Against Localization Geometries

**Figure 14:**
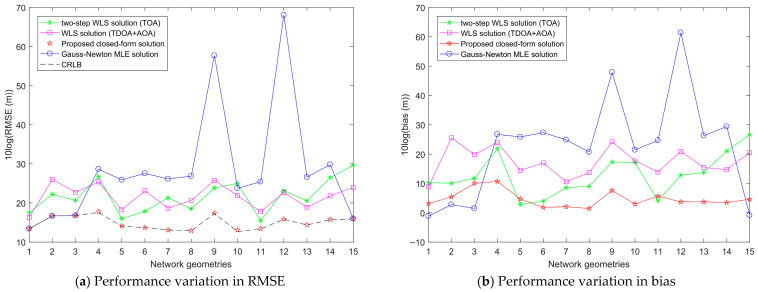
Performance variation in various target localization methods on 15 random network geometries.

To evaluate the robustness of various localization methods against localization geometry, 15 network geometries and target positions are randomly generated to simulate the target localization performance of each method. For each localization geometry, $\sigma_{s}^{2}$, $\sigma_{r}^{2}$ and $\sigma_{A}^{2}$ are fixed at 10^2^ m^2^, 10^2^ m^2^ and 1 deg^2^, respectively. The results are shown in Figure 14.

Figure 14a shows the RMSE variation in each target localization method across 15 random localization geometries. It can be seen that regardless of the localization geometry, the RMSE of the proposed closed-form solution is lower than those of the other three methods, achieving the CRLB accuracy with the smallest RMSE variation. The RMSEs of the Gauss-Newton MLE solution, the two-step WLS solution, and the WLS solution all exhibit larger variations. Notably, the Gauss-Newton MLE solution reaches the CRLB accuracy in four localization geometries, while in the remaining geometries, its RMSE variation is the largest. In some localization geometries, the RMSE of the Gauss-Newton MLE solution even increases abruptly to very high values, leading to localization failure. Figure 14b presents the bias variation in each target localization method across 15 random localization geometries. It can be seen that, except for the localization geometries where the Gauss-Newton MLE solution achieves the CRLB accuracy, the bias of the proposed closed-form solution is the smallest in the other localization geometries, with the smallest bias variation. In most network geometries, the bias of the Gauss-Newton MLE solution is the largest and exhibits significant variation.

From the above analysis, it is apparent that localization geometry has the least impact on the target localization performance of the proposed closed-form solution, indicating that the proposed closed-form solution has the best robustness against localization geometry. The Gauss-Newton MLE solution has the worst robustness and achieves excellent localization performance only in certain localization geometries, which can be used for target localization in specific localization geometries.

Based on the simulations and analyses above, we can see that within the tested noise range, the proposed closed-form solution has the best localization performance, achieving the CRLB accuracy below a large noise threshold. The proposed closed-form solution has the best robustness, and its localization performance is less affected by localization geometry. In addition, the Gauss-Newton MLE solution achieves the CRLB accuracy and has the lowest bias in some specific localization geometries, making it suitable for target localization in those specific localization scenarios.

## 7. Conclusions

This paper investigated the target localization method based on TOA and AOA measurements to improve the localization accuracy of the cooperative target in the presence of sensor position errors and measurement noise. First, for the deep-sea target localization scenario, the paper proposed a slant range estimation method based on sound ray bending correction to improve the accuracy of range measurement. Then, we proposed a closed-form solution based on the two-step WLS. To further reduce the localization bias of the target, this paper also derived a Gauss-Newton MLE solution. Subsequently, we derived the CRLB of target localization in the localization scenario and simulated the localization performance of the proposed method and other methods over 100 random localization geometries. Simulation results show that the proposed closed-form solution can achieve the CRLB accuracy and improve the localization accuracy of the target compared to other localization methods. Additionally, the Gauss-Newton MLE method can achieve the CRLB accuracy and minimize the localization bias of the target in certain localization geometries, which is suitable for target localization in specific localization scenarios.

## Data Availability

The original contributions presented in this study are included in the article. Further inquiries can be directed to the corresponding author.

## Abbreviations

The following abbreviations are used in this manuscript:

UASNs	Underwater acoustic sensor networks
TOA	Time of arrival
AOA	Angle of arrival
CRLB	Cramér-Rao lower bound
RMSE	Root mean square error
WLS	Weighted least squares
MLE	Maximum likelihood estimation

## Appendix A

$d^{o}={\left[\kappa^{oT},s^{oT}\right]}^{T}$ and $\lambda^{o}={\left[u^{oT},s^{oT}\right]}^{T}$, we have

(A1) $$B_{1}=\frac{\partial d^{o}(\lambda^{o})}{\partial \lambda^{oT}}{|}_{\lambda^{o}=\lambda^{\left(1\right)}}=\left[\begin{array}{cc} \frac{\partial \kappa^{o}}{\partial u^{oT}} & \frac{\partial \kappa^{o}}{\partial s^{oT}}\\ \frac{\partial s^{o}}{\partial u^{oT}} & \frac{\partial s^{o}}{\partial s^{oT}} \end{array}\right]$$

where

$$\frac{\partial \kappa^{o}}{\partial u^{oT}}={\left(\frac{\partial \kappa^{oT}}{\partial u^{o}}\right)}^{T},\;\frac{\partial \kappa^{o}}{\partial s^{oT}}={\left(\frac{\partial \kappa^{oT}}{\partial s^{o}}\right)}^{T},\;\frac{\partial s^{o}}{\partial u^{oT}}=O_{3M\times 3},\;\frac{\partial s^{o}}{\partial s^{oT}}=I_{3M\times 3M}.$$

Substituting (42) and the initial value of $\lambda^{o}$ into (A1), the value of $B_{1}$ can be obtained.

## Appendix B

In (42), let $C=\left[c_{1},c_{2},\cdots ,c_{M}\right]$, $D=\left[D_{1},D_{2},\cdots ,D_{M}\right]$, $E=[e_{1},e_{2},\cdots ,e_{M}]$, $F=[F_{1},F_{2},\cdots ,F_{M}]$. Substituting them into $F_{\kappa }$, $F_{r}$, and $F_{a}$ yields

(A2) $$\begin{array}{l} F_{\kappa }=\left[\begin{array}{cc} C & D\\ E & F \end{array}\right]\left[\begin{array}{cc} Q_{r}^{-1} & O\\ O & Q_{a}^{-1} \end{array}\right]{\left[\begin{array}{cc} C & D\\ E & F \end{array}\right]}^{T}\\ \;\;\;\;\;=\left[\begin{array}{cc} CQ_{r}^{-1}C^{T} & CQ_{r}^{-1}E^{T}\\ EQ_{r}^{-1}C^{T} & EQ_{r}^{-1}E^{T} \end{array}\right]+\left[\begin{array}{cc} DQ_{a}^{-1}D^{T} & DQ_{a}^{-1}F^{T}\\ FQ_{a}^{-1}D^{T} & FQ_{a}^{-1}F^{T} \end{array}\right] \end{array}$$

(A3) $$F_{r}=\left[\begin{array}{c} C\\ E \end{array}\right]Q_{r}^{-1}{\left[\begin{array}{c} C\\ E \end{array}\right]}^{T}=\left[\begin{array}{cc} CQ_{r}^{-1}C^{T} & CQ_{r}^{-1}E^{T}\\ EQ_{r}^{-1}C^{T} & EQ_{r}^{-1}E^{T} \end{array}\right]$$

(A4) $$F_{a}=\left[\begin{array}{c} D\\ F \end{array}\right]Q_{a}^{-1}{\left[\begin{array}{c} D\\ F \end{array}\right]}^{T}=\left[\begin{array}{cc} DQ_{a}^{-1}D^{T} & DQ_{a}^{-1}F^{T}\\ FQ_{a}^{-1}D^{T} & FQ_{a}^{-1}F^{T} \end{array}\right]$$

From (A2)–(A4), it can be seen that $F_{\kappa }{=F}_{r}+F_{a}$.

## Appendix C

For $F_{r}={\left(\frac{\partial r^{o}}{\partial \lambda^{oT}}\right)}^{T}Q_{r}^{-1}\frac{\partial r^{o}}{\partial \lambda^{oT}}$, $Q_{r}^{-1}$ is a positive definite matrix. Given any 3(M+1)×1 nonzero vector d, we have

(A5) $$d^{T}F_{r}d={\left(\frac{\partial r^{o}}{\partial \lambda^{oT}}d\right)}^{T}Q_{r}^{-1}\left(\frac{\partial r^{o}}{\partial \lambda^{oT}}d\right)$$

For the term ${\left(\frac{\partial r^{o}}{\partial \lambda^{oT}}d\right)}^{T}$, we have

(A6) $${\left(\frac{\partial r^{o}}{\partial \lambda^{oT}}d\right)}^{T}=d^{T}\frac{\partial r^{oT}}{\partial \lambda^{o}}=d^{T}\left[\begin{array}{cccc} \frac{u^{o}-s_{1}^{o}}{r_{1}^{o}} & \frac{u^{o}-s_{2}^{o}}{r_{2}^{o}} & \cdots & \frac{u^{o}-s_{M}^{o}}{r_{M}^{o}}\\ -\frac{u^{o}-s_{1}^{o}}{r_{1}^{o}} & 0_{3} & \cdots & 0_{3}\\ 0_{3} & -\frac{u^{o}-s_{2}^{o}}{r_{2}^{o}} & \cdots & 0_{3}\\ \vdots & \vdots & \ddots & \vdots \\ 0_{3} & 0_{3} & \cdots & -\frac{u^{o}-s_{M}^{o}}{r_{M}^{o}} \end{array}\right]$$

If $d^{T}=k_{scale}\left[\begin{array}{cccc} 1 & 1 & \cdots & 1 \end{array}\right]$, where $k_{scale}$ is a non-zero constant, then ${\left(\frac{\partial r^{o}}{\partial \lambda^{oT}}d\right)}^{T}=d^{T}\frac{\partial r^{oT}}{\partial \lambda^{o}}=0^{T}$. For any other non-zero vector, ${\left(\frac{\partial r^{o}}{\partial \lambda^{oT}}d\right)}^{T}=d^{T}\frac{\partial r^{oT}}{\partial \lambda^{o}}\neq 0^{T}$. Since $Q_{r}^{-1}$ is a positive definite matrix, it follows that

(A7) $$d^{T}F_{r}d={\left(\frac{\partial r^{o}}{\partial \lambda^{oT}}d\right)}^{T}Q_{r}^{-1}\left(\frac{\partial r^{o}}{\partial \lambda^{oT}}d\right)\geq 0$$

For any non-zero vector d, $d^{T}F_{r}d\geq 0$. Thus

(A8) $$F_{r}≽O$$

Similarly, it can be proven that $F_{a}≽O$.
